# Mathematical Modeling for Inherited Diseases

**DOI:** 10.1155/2017/1975780

**Published:** 2017-07-09

**Authors:** Saima Anis, Madad Khan, Saqib Khan

**Affiliations:** ^1^Department of Mathematics, COMSATS Institute of Information Technology, Abbottabad, Pakistan; ^2^Department of Mathematics, Government Postgraduate College, Abbottabad, Pakistan

## Abstract

We introduced a new nonassociative algebra, namely, left almost algebra, and discussed some of its genetic properties. We discussed the relation of this algebra with flexible algebra, Jordan algebra, and generalized Jordan algebra.

## 1. Introduction

We introduced a new nonassociative and noncommutative algebra which has several properties similar to nonassociative and commutative algebras. The relation of the left almost algebra with other nonassociative algebras is useful and interesting to be known; in this regard, we found some relations with known algebras, namely, flexible, alternative, and Jordan algebras. We discussed some characteristics of this algebra which is similar to a commutative and associative algebra. We discussed some of the genetic properties of this algebra.

*Some Basics from Genetics*. Here, we discuss some simple ideas in genetics for nongeneticists and for those who are purely related to mathematics. Each cell of an organism contains long thread-like structures called chromosomes which are located inside the nucleus of animals and plants. Chromosomes are made of protein and a single molecule of deoxyribonucleic acid (DNA). After cell division, chromosomes pass from parent cells to the newborn cells. The particular intersections that the DNA carries make each living creature unique from others. The molecules of the DNA are too long, which can be fitted inside the cells only by chromosomes. Moreover, chromosomes play an important role in copying and distributing DNA accurately in the whole process of cell division. Problems in chromosomes in new cells can create serious issues like leukemia and some types of cancer. Males and females have different chromosomes; that is, females have two *X* chromosomes in their cells whereas males have one *X* and one *Y* chromosome. Humans have 23 pairs of chromosomes, with a total of 46 chromosomes. A gene is a unit of hereditary information and lies on chromosomes. A gene can take different forms which are called alleles. In these 23 pairs, one represents the sexual character in males; a locus which occurs on 22 pairs of chromosomes is called autosomal, whereas a locus on one pair is called sex-linked. Diploid cells are those which carry a double set of chromosomes and haploid cells are those which carry a single set of chromosomes. Diploid cells produce sex cells called gametes through a process called meiosis. Mitosis is the process of reproduction in haploid cells. The gametes cells meet each other and give a zygote which is again a diploid cell.

*Basics of Genetic Algebra.* After the theory of Charles Darwin, it was Gregor Mendel who studied the natural laws of genetic inheritance and tried to express them in a mathematical language. In [1–3], Etherington introduced a new method of nonassociative algebras to study genetics. Holgate studied these algebras with genetic realizations in [4–7]. Reed discussed the nonassociative algebraic structure of genetic inheritance in [8]. The genetic algebra with mutation has been discussed in [9, 10]. Several other authors studied nonassociative algebras with genetic realizations (for details, see [10–14]).

Let *R* be a nonempty set together with two binary operations “+” and “·” which satisfies all the axioms of an associative ring (algebra) except an associative property with respect to multiplication; then, it is known as a nonassociative ring (algebra). Lie ring was introduced by defining and replacing a new binary operation [*a*, *b*] = *ab* − *ba*, for all *a*, *b*, with ordinary multiplication of an associative ring (algebra); obviously, it is a nonassociative ring (algebra). By defining a new binary operation *a* · *b* = (1/2)(*ab* + *ba*) on an associative algebra over a field whose characteristic is not equal to 2, we obtain another important nonassociative algebra known as Jordan algebra. It is worth mentioning here that the theory of nonassociative algebras is a fruitful branch of algebra. Most importantly, the class of nonassociative algebras has closed connections with other branches of mathematics; also, it has closed connections with quantum mechanics, physics, biology, and other sciences. The crucial part of this theory is the theory of nearly associative rings and algebras: Lie, alternative, and Jordan algebras. In short, considering nonassociative algebras over real number fields has several applications in biology especially in genetics. Moreover, there are some other classes of nonassociative algebras closely related to genetics which are popular among mathematicians and geneticists for their usefulness in genetics. Generally, these types of algebras are commutative and nonassociative. In fact, one can study the properties of genetics by making the mathematical models using nonassociative, commutative algebras. To visualize the concept of such algebras, let us pay attention to some specific classes of algebras like gametic, zygotic, and copular algebras.

Here, we will discuss some simple algebras with genetic realizations. In order to understand the algebraic properties in genetics, simple Mendelian inheritance has been considered in [10]. If we consider a single gene with two alleles *x*_1_ and *x*_2_, after mating of alleles *x*_1_ and *x*_2_, we get the zygotes *x*_1_*x*_1_, *x*_1_*x*_2_, *x*_2_*x*_1_, and *x*_2_*x*_2_, where *x*_1_*x*_1_, *x*_2_*x*_2_ are known as homozygotes while *x*_1_*x*_2_, *x*_2_*x*_1_ are called heterozygotes. In particular, *x*_1_*x*_2_ = *x*_2_*x*_1_, which means that the commutative law holds in this case, which genetically represents the notion that mating of allele *x*_1_ with allele *x*_2_ is the same as mating of *x*_2_ with *x*_1_. Let us consider the equation *x*_1_*x*_2_ = (1/2)(*x*_1_ + *x*_2_). This linear equation represents the notion that *x*_1_*x*_1_ = *x*_1_ and *x*_2_*x*_2_ = *x*_2_. The algebra with the multiplication table above is known as gamete algebra. Moreover, the zygotic algebra can be obtained from the gametic algebra by commutative duplication. From mathematical perspectives, the juxtaposition between *x*_1_ and *x*_2_ shows a binary operation which is not associative because (*x*_1_*x*_2_)*x*_2_ ≠ *x*_1_(*x*_2_*x*_2_) or equivalently (*x*_1_*x*_2_)*x*_2_ = *x*_1_(*x*_2_*x*_2_) says that both alleles *x*_1_ and *x*_2_ are the same. Therefore, the associative law does not hold for the gametic algebra and it also does not hold for the zygotic algebra. Generally, algebras associated with genetics are commutative but nonassociative. In [3], Etherington proved that the zygotic algebra can be obtained from the gametic algebra through a commutative duplication process. Now, it is interesting to note that there is a class of algebras which is nonassociative and noncommutative but possesses many characteristics similar to commutative and associative algebras and has close relations with commutative algebras. Using notions from these algebras, we give a more general definition of gametic and zygotic algebras. Moreover, this nonassociative and noncommutative algebra has closed connections with Jordan and flexible algebras. In addition to this, if it contains a left identity, then it satisfies the famous Jordan identity and the generalized Jordan identity. This algebra works mostly like a commutative algebra; for instance, *x*^2^*y*^2^ = *y*^2^*x*^2^, for all *x*, *y*, holds in a commutative algebra while this equation also holds for a left almost algebra, and if a left almost algebra contains a left identity *e*, then *xy* = (*yx*)*e*, for any *x*, *y*. In fact, the structure is nonassociative and noncommutative but it possesses many properties which usually hold in associative and commutative algebraic structures. Also, defining a new operation on this algebra gives a commutative and associative algebra.

In this paper, we will discuss those algebras which are nonassociative and noncommutative but have close connections with nonassociative and commutative algebras. We generalized the definition available in [8] and introduced a new generalized definition for gamete algebras. Moreover, we introduce a new class of a nonassociative algebra called left almost algebra.

We restrict ourselves by considering a gene on a particular locus on a chromosome. Here, we begin with the definition of gametic algebra [8]. Consider *n* gametes *a*_1_, *a*_2_,…, *a*_*n*_ as basis elements of an *n*-dimensional real vector space. Multiplication is defined by

(1) aiaj=∑k=1nγijkak,such  that  0≤γijk≤1,  i,j,k=1,2,…,n,  ∑k=1nγijk=1,  i,j,k=1,2,…,n,  γijk=γjik,  i,j,k=1,2,…n,

where *γ*_*ijk*_ represent the relative gene frequencies.

The resulting algebra *𝔖* is called an *n*-dimensional gametic algebra.

*Some Basic Notions of Nonassociative Algebras*. A groupoid *S* is called a left almost semigroup if it satisfies the following left invertive law, (*ab*)*c* = (*cb*)*a*, for all *a*, *b*, *c* ∈ *S*. Holgate called it a left invertive groupoid [4]. An element *e* of a groupoid *S* is called left (right) identity if *ex* = *x*(*xe* = *x*) for all *x* in *S*. A left identity in a left almost semigroup is unique.

Let *a*, *b*, *c* ∈ *S*, where *S* is a left almost semigroup with left identity *e*. Then,

(2) $$\begin{array}{c} a\left(\;bc\;\right)=b\left(\;ac\;\right),\;\forall a,b,c\in S. \end{array}$$

But the converse of the above statement is not true.

If a left almost semigroup contains a right identity, then it becomes a commutative semigroup. A left almost semigroup *S* is a mid structure between a groupoid and a commutative semigroup.

From the discussion above, it is easy to conclude that this nonassociative structure with left identity has a closed connection with a commutative semigroup.

A nonassociative algebra *𝔄* is a vector space over a field *𝔉* along with the bilinear multiplication from *𝔄* × *𝔄* → *𝔄*, satisfying the following distributive properties:

(3) αa+βbc=αac+βbc,aαb+βc=αab+βac,∀α,β∈F,  a,b,c∈A.

A nonassociative algebra *𝔄* is called left almost algebra over a field *𝔉* if it satisfies the left invertive property with respect to multiplication.

Several authors discussed mostly commutative and nonassociative algebras with genetic realizations. There are some cases of noncommutative, nonassociative algebraic structures which were discussed in [2, 10, 14]. Moreover, Mendel's algebra is interesting to discuss. To study such cases of Mendel's algebra with mutation, we introduce a new class of nonassociative and noncommutative algebras known as left almost algebras. These algebras possess many characteristics which are similar to those of commutative nonassociative algebras with genetic realizations. Here, we give the generalized definition for gametic algebra; consider *n* gametes *a*_1_, *a*_2_,…, *a*_*n*_ as basis elements of an *n*-dimensional left almost vector space over *ℝ*. If we define multiplication by

(4) aiaj=γij1a1+γij2a2+γij3a3+⋯+γijnan,such  that  1=γij1+γij2+γij3+⋯+γijn,  i,j,k=1,2,…,n,

where  0 ≤ *γ*_*ijk*_ ≤ 1  for all *i*, *j*, *k* = 1,2,…, *n*,  *γ*_*ijk*_ represent the relative gene frequencies.

Then, the resulting algebra *𝔄* is called an *n*-dimensional nonassociative and noncommutative left almost gametic algebra.

Let (*a*_1_,…, *a*_*n*_) be a basis representing the *n* alleles generating a gametic left almost algebra and the multiplication defined as *a*_*i*_*a*_*j*_ = (1/2)(*a*_*i*_ + *a*_*j*_). Consider the mapping *ω* : *𝔙* → *ℝ* and let *w* be the weight function defined as *ω*(*a*_*i*_) = 1. For any element *x* of *𝔄*, *x* = ∑*α*_*i*_*a*_*i*_. Thus,

(5) x2xx=∑i=1nαiai∑i=1nαiai=∑i=1n ∑i=1nαiaiαiai=∑i=1n ∑i=1nαiαiaiai=∑i=1n ∑i=1nαiαiai=∑i=1n ∑i=1nαiαiai=∑i=1nαi∑i=1nαiai=x,Provided  ∑i=1nαi=1.

Now, in a similar way, we can define the zygotic algebra and for this we consider *n* gametes *a*_*ij*_ = *a*_*i*_*a*_*j*_ (considering only *i* ≤ *j*), and then random mating of zygotes *a*_*ij*_ with *a*_*pq*_ will produce zygotes *a*_*ks*_ with a particular ratio, say *γ*_*ij*,*pq*,*ks*_. Thus, we define the following generalized zygotic multiplication as

(6) $$\begin{array}{c} a_{ij}a_{pq}=\underset{k\leq s}{\sum }\gamma_{ij,pq,ks}a_{ks},\;\text{such that }\underset{k\leq s}{\sum }\gamma_{ij,pq,ks}=1,\;\;i,j,k=1,2,\ldots ,n\text{ such that }0\leq \gamma_{ij,pq,ks}\leq 1\;\;\forall i,j,k=1,2,\ldots ,n, \end{array}$$

where *γ*_*ij*,*pq*,*ks*_ are the relative gene frequencies.

An element *x* in our noncommutative, nonassociative algebra *𝔄* indicates a population or a gene pool and it can be expressed as a linear combination of the basis elements *a*_1_, *a*_2_,…, *a*_*n*_ as *x* = *λ*_1_*a*_1_ + *λ*_2_*a*_2_ + *λ*_3_*a*_3_ + ⋯+*λ*_*n*_*a*_*n*_ and *λ*_1_ + *λ*_2_ + *λ*_3_ + ⋯+*λ*_*n*_ = 1, where *λ*_*l*_ ∈ *𝔉*, for all *l* = 1,2,…, *n*.

The algebra with genetic realization arising from expression (4) is more general than the one arising from (1) because the algebra is both nonassociative and noncommutative.

An algebra *𝔄* is called flexible if it satisfies the following property:

(7) $$\begin{array}{c} \left(\;xy\;\right)x=x\left(\;yx\;\right),\;\forall x,y\text{ in }A. \end{array}$$

An algebra *𝔄* is called a generalized Jordan algebra if it satisfies the following property:

(8) $$\begin{array}{c} \left(\;xy\;\right)\left(\;xx\;\right)=x\left(\;y\left(\;xx\;\right)\;\right),\;\forall x,y\text{ in }A. \end{array}$$

It is obvious that both flexible and generalized Jordan algebras are different but if *x* = *x*^2^ for all *x* then both become identical.

## 2. Mutation Algebra

In [9], the author considered mutation algebra with mutation rates *r* and *s*, the gametic algebra having the basis with elements *D* and *R*, where the multiplication table is defined as

(9) D2=1−rD+rR,

(10) DR=121−r+sD+121−s+rR.

Then, the author chose another basis with elements *a* = *D* and *b* = *D* − *R*, and thus

(11) a2=a−rb,ab=121−r−sb,b2=0.

Let us define an abstract algebra *𝔄* generated by {*a*_*i*_ : 1 ≤ *i* ≤ *n*} over a finite field *𝔉*. If we define the binary operation “⋆” on *𝔄* as *a*_*i*_⋆*a*_*j*_ = *αa*_*i*_ + *α*^2^*a*_*j*_(*α* + *α*^2^ = 1), then this algebra satisfies (10) but *a*_*i*_⋆*a*_*i*_ = *a*_*i*_. Therefore, it is important to mention here that the algebra defined by this operation is not totally consistent with the mutation algebra introduced by Gonshor above. This simply implies that there is a lack of one hundred correspondences between this algebra and the algebra defined in (10) but there are still several similarities existing between the ideas of the mutation algebra and the algebra we introduced. In the next theorem, we will prove that this algebra is a left almost algebra. We denote this algebra by *ℳ*_*𝔫*_(*α*_*𝔉*_).

**Theorem 1 .**

*ℳ*_*𝔫*_(*α*_*𝔉*_) is a noncommutative and nonassociative left almost algebra.

**Proof**

Obviously, *ℳ*_*𝔫*_(*α*_*𝔉*_) is closed. Next, we will show that *ℳ*_*𝔫*_(*α*_*𝔉*_) satisfies the left invertive property, for this left *X*, *Y*, *Z* ∈ *ℳ*_*𝔫*_(*α*_*𝔉*_). Then, *X* = ∑_*j*=1_^*n*^*β*_*j*_*a*_*j*_, *Y* = ∑_*k*=1_^*n*^*γ*_*k*_*a*_*k*_ and *Z* = ∑_*l*=1_^*n*^*δ*_*l*_*a*_*l*_. To prove (*X*⋆*Y*)⋆*Z* = (*Z*⋆*Y*)⋆*X*, we need to prove (*a*_*j*_⋆*a*_*k*_)⋆*a*_*l*_ = (*a*_*l*_⋆*a*_*k*_)⋆*a*_*j*_.

(12) aj⋆ak⋆alααaj+α2ak+α2al=α2aj+α3ak+α2al,al⋆ak⋆ajααal+α2ak+α2aj=α2al+α3ak+α2aj=α2x+α3y+α2z.

It is not associative because

(13) aj⋆ak⋆alαaj+α2αak+α2al=αaj+α3ak+α4al.

Moreover, *a*_*j*_⋆*a*_*k*_ = *αa*_*j*_ + *α*^2^*a*_*k*_ and *a*_*k*_⋆*a*_*j*_ = *αa*_*k*_ + *α*^2^*a*_*j*_. Thus, *XY* ≠ *YX*, for some *X*, *Y*.

If we consider this theorem for a finite field of cardinality 4 which is the extension of *ℤ*_2_, then *t*^2^ + *t* = 1 gives *t*^2^ + *t* + 1 = 0. More generally, *x*^2^ + *x* + 1 = 0 is the quadratic equation representing the irreducible polynomial in *ℤ*_2_ = {0,1}. Thus, *GF*(2^2^) = {0,1, *t*, *t*^2^}, and thus *GF*(2^2^)∖{0} = *𝔉*∖{0} = 〈*t* : *t*^3^ = 1〉 = {1, *t*, *t*^2^}. We denote this algebra by *ℳ*_4_(*𝔱*_*𝔉*_). Roots of the equation *x*^2^ + *x* = 1 are $\left(-1+\sqrt{5}\right)/2$ and $\left(-1-\sqrt{5}\right)/2$. Thus,

(14) ak⋆aj=αak+α2ajα+α2=1,  where  α=−1±52.

Obviously, *a*_*j*_⋆*a*_*j*_ = *αa*_*j*_ + *α*^2^*a*_*j*_ = (*α* + *α*^2^)*a*_*j*_ = *a*_*j*_.

**Theorem 2 .**

The algebra *ℳ*_4_(*𝔱*_*𝔉*_) is a generalized Jordan algebra.

**Proof**

Clearly, *ℳ*_4_(*𝔱*_*𝔉*_) is a left almost algebra. Next, we will prove that it satisfies the property of flexible algebra. We have *a*_*j*_⋆*a*_*j*_ = *αa*_*j*_ + *α*^2^*a*_*j*_ = (*α* + *α*^2^)*a*_*j*_ = *a*_*j*_. Therefore,

(15) X⋆X∑j=1nβjaj⋆∑j=1nβjaj=∑j=1n ∑j=1nβjβjaj⋆aj=∑j=1n ∑j=1nβjβjaj=∑j=1nβj∑j=1nβjaj=∑j=1nβjaj.

Thus,

(16) X⋆Y⋆X⋆XX⋆X⋆Y⋆X=Y⋆X⋆X⋆X=X⋆X⋆Y⋆X=X⋆Y⋆X=X⋆Y⋆X⋆X.

**Corollary 3 .**

The algebra *ℳ*_4_(*𝔱*_*𝔉*_) is a flexible algebra.

It is obvious from Theorem 2 that *ℳ*_4_(*𝔱*_*𝔉*_) contains idempotent elements and we know that idempotent elements in nonassociative algebras have their own importance. Thus, we arrived at the following remark.

**Remark 4 .**

From the biological point of view, the idempotents in the algebra *ℳ*_4_(*𝔱*_*𝔉*_) have their own usefulness. Since this algebra has several characteristics similar to a nonassociative algebra arising in genetics, the idempotent elements of this algebra may be used for equilibria of a population described by some nonassociative algebras with genetic realizations.

In the following, we will consider some other nonassociative algebras and will discuss their relations with the left almost algebra mathematically.

An alternative algebra *𝔄* is a nonassociative algebra satisfying the following properties:

(17) xxy=xxy,yxx=yxx,∀x,y  in  A.

**Lemma 5 .**

If *𝔄* is a left almost alternative algebra, then *x*(*xy*) = (*xx*)*y* = (*yx*)*x* = *y*(*xx*) and *x*^2^*y* = *yx*^2^, for all *x* and *y* in *𝔄*.

**Proof**

Let *x* and *y* belong to *𝔄*; then, *x*(*xy*) = (*xx*)*y* = (*yx*)*x* = *y*(*xx*), so *x*^2^*y* = *yx*^2^, for all *x* and *y*.

It is proved above that *x*^2^*y* = *yx*^2^ for all *x* and *y* in *𝔄*. Thus, *x*^2^*x* = *xx*^2^ for *x* in *𝔄*. Therefore, we can define powers of an element in *𝔄*.

**Lemma 6 .**

If *𝔄* is a left almost alternative algebra that contains a left identity *𝔢*, then *𝔄* becomes commutative and associative with identity.

**Proof**

Since *x*^2^*y* = *yx*^2^, for all *x* and *y*, therefore *x* = *𝔢x* = *𝔢*^2^*x* = *x𝔢*^2^ = *x𝔢*. Then,

(18) $$\begin{array}{c} xy=\left(\;xy\;\right)e=\left(\;ey\;\right)x=yx. \end{array}$$

It is easy to see that commutativity and the left invertive law give associativity.

For the rest of the paper, by *𝔄*, we shall mean the left almost alternative algebra satisfying (2).

**Lemma 7 .**

*yx* ^*n*^ = *x*^*n*^*y*, for all *x*, *y* in *𝔄* and for *n* ≥ 2.

**Proof**

We already proved that *x*^2^*y* = *yx*^2^, for all *x*, *y* in *𝔄*. Then,

(19) x2yxyx2x=x2yx=xyx2=x2yx=yx2x=yx3,yx2xxx2y=x3y.

Thus, *yx*^3^ = *x*^3^*y*. Let us assume that let *yx*^*k*^ = *x*^*k*^*y*, for *k* ≥ 3. Then,

(20) xkyx=xyxk=xkyx=yxkx=yxk+1,yxkx=xxky=xk+1y.

**Theorem 8 .**

Every *𝔄* becomes a generalized Jordan algebra.

**Proof**

Let *x*, *y* ∈ *𝔄*. Then,

(21) $$\begin{array}{c} \left(\;xy\;\right)x^{2}=x^{2}\left(\;yx\;\right)=y\left(\;x^{2}x\;\right)=y\left(\;xx^{2}\;\right)=x\left(\;yx^{2}\;\right). \end{array}$$

Hence, (*xy*)*x*^2^ = *x*(*yx*^2^).

**Lemma 9 .**

(*x*^2^*y*)*x* = *x*^2^(*yx*), for all *x*, *y* in *𝔄*.

**Proof**

Using *x*^2^*y* = *yx*^2^, we get

(22) $$\begin{array}{c} \left(\;x^{2}y\;\right)x=\left(\;xy\;\right)x^{2}=x^{2}\left(\;xy\;\right)=\left(\;yx\;\right)x^{2}=x^{2}\left(\;yx\;\right). \end{array}$$

We get a more generalized form of generalized Jordan algebra which is available in the following crucial theorem.

**Theorem 10 .**

(*x*^2^*y*)*z* = *x*^2^(*yz*), *x*^2^(*yz*) = *x*^2^(*zy*), for all *x*, *y*, *z* in *𝔄*.

**Proof**

Using *x*^2^*y* = *yx*^2^, we get

(23) $$\begin{array}{c} \left(\;x^{2}y\;\right)z=\left(\;zy\;\right)x^{2}=x^{2}\left(\;zy\;\right)=\left(\;yz\;\right)x^{2}=x^{2}\left(\;yz\;\right). \end{array}$$

This theorem represents a mathematical model showing that mating of *x*^2^ with *yz* is the same as mating of *x*^2^ with *zy*.

**Proposition 11 .**

(*ab*)(*cd*) = (*db*)(*ca*) = (*dc*)(*ba*), for all *a*, *b*, *c*, *d* in *𝔄*.

**Proof**

The proof is easy.

For any *a* in *S*, we put *a*^1^ = *a* and *a*^*n*+1^ = *a*^*n*^*a*, where *n* is a positive integer.

**Proposition 12 .**

*𝔄* has associative powers.

**Proof**

The proof is easy.

**Proposition 13 .**

*a* ^*m*^ *a* ^*n*^ = *a*^*m*+*n*^, for all *a* ∈ *𝔄* and positive integers *m*, *n*.

**Proof**

According to Proposition 12, the result is true for *m*⩾1. Again, by Lemma 7, we obtain

(24) am+1anamaan=aaman=amana=am+na=am+n+1.

Hence, *a*^*m*^*a*^*n*^ = *a*^*m*+*n*^  ∀*a* ∈ *𝔄*.

**Proposition 14 .**

(*a*^*m*^)^*n*^ = *a*^*mn*^ for all *a* ∈ *A* and positive integers *m*, *n*.

**Proof**

The result is true for *n* = 1. Suppose it is true for *n* > 1. Then, we obtain

(25) $$\begin{array}{c} {\left(\;a^{m}\;\right)}^{n+1}={\left(\;a^{m}\;\right)}^{n}a^{m}=a^{mn}a^{m}=a^{mn+m}=a^{m\left(n+1\right)}. \end{array}$$

Hence, by induction on *n*, (*a*^*m*^)^*n*^ = *a*^*mn*^ for all *a* in *S* and positive integers *m*, *n*.

**Proposition 15 .**

(*ab*)^*n*^ = *a*^*n*^*b*^*n*^, for all *a*, *b* in *𝔄* and for positive integer *n* ≥ 1.

**Proof**

The result is true for *n* = 1. If *n* = 2, then

(26) $$\begin{array}{c} {\left(\;ab\;\right)}^{2}=\left(\;ab\;\right)\left(\;ab\;\right)=\left(\;aa\;\right)\left(\;bb\;\right)=a^{2}b^{2}. \end{array}$$

Suppose that the result is true for *n* = *k*. Then, we get

(27) abk+1abkab=akbkab=akabkb=ak+1bk+1.

Hence, the result is true for all positive integers.

**Theorem 16 .**

*x* ^*n*^ *y* ^*m*^ = *y*^*m*^*x*^*n*^, for *n* ≥ 1, *m* ≥ 2, for all *x*, *y* in *𝔄*.

**Proof**

The proof follows from Lemma 7.

**Theorem 17 .**

(*x*^*n*^*x*^*m*^)*y* = *y*(*x*^*m*^*x*^*n*^), for *m* ≥ 2, *n* ≥ 1.

**Proof**

Let *x*, *y* ∈ *𝔄*. Then,

(28) xnxmyxmxny=xnyxm=xmyxn=yxmxn.

**Theorem 18 .**

Every *𝔄* satisfies the generalized Jordan identity (*x*^*m*^*y*)*x*^*n*^ = *x*^*m*^(*yx*^*n*^) for *m* ≥ 1, *n* ≥ 2.

**Proof**

We will use induction. For *m* = 1 and *n* = 2, it is the same as in Theorem 17.

(29) xmyxnyxmxn=xnxmy=yxmxn=xmyxn.

It is obvious from the above that the left almost algebra has closed connections with generalized Jordan algebra and flexible algebra.

It is interesting to note that genetic algebras (gametic, zygotic, copular, train, Bernstein, etc.) do not satisfy the properties of Jordan algebra but the left almost algebra becomes a Jordan algebra. Moreover, this algebra is power associative and satisfies the generalized identity (*x*^*m*^*y*)*x*^*n*^ = *x*^*m*^(*yx*^*n*^) introduced by Jordan. It also satisfies *x*^*n*^*y*^*m*^ = *y*^*m*^*x*^*n*^ for *m*, *n* ≥ 2. This algebra is the generalization of the Jordan algebra. It is noncommutative and nonassociative but possesses many characteristics similar to Jordan algebra. Since Jordan algebra has many applications in genetics, it is concluded that our new generalized algebra will give direction for applications in genetics.

## 3. Conclusion

In this paper, we introduced a new nonassociative and noncommutative algebra with genetic realizations. We discussed its link with flexible, alternative, and Jordan algebras. This algebra possesses many characteristics similar to a commutative and associative algebra. We discussed some of the genetic properties of this algebra. In our future work, we will focus on some other nonassociative algebras. We conclude that this research will give a new direction for applications in genetics.
